# Impact of semen extenders, storage duration, and insemination timing on semen quality and reproductive performance in Palestinian Assaf sheep

**DOI:** 10.14202/vetworld.2025.808-818

**Published:** 2025-04-07

**Authors:** Wael Halawa, Samia Khnissi, Ikram Bensouf, Bochra Bejaoui, Hela Chalouati, Muayad Salman, Naceur M’Hamdi

**Affiliations:** 1 Animal and Food Genetic Resources Research Laboratory, LR15AGRO01; National Agronomic Institute of Tunisia, University of Carthage, 43 Av. Charles Nicolle, Tunis, 1082 Tunisia; 2 National Agricultural Research Center, Palestinian Ministry of Agriculture, Ramallah, West Bank, Palestine; 3 Laboratory of Animal and Forage Production, National Institute of Agronomic Research of Tunisia, University of Carthage, Tunis, Tunisia; 4 National Institute of Research and Pysico-chemical analysis (INRAP), Laboratory of Useful Materials, Technopark of Sidi Thabet, Ariana 2020, University of Carthage, Tunis, Tunisia; 5 Department of Chemistry, Faculty of Sciences of Bizerte, 7021, Zarzouna, Bizerte, University of Carthage, Tunis, Tunisia; 6 Palestinian Ministry of Agriculture, Ramallah, West Bank, Palestine

**Keywords:** artificial insemination, breeding efficiency, Palestinian Assaf sheep, semen preservation

## Abstract

**Background and Aim:**

Artificial insemination (AI) is a critical technique in sheep breeding programs, yet its success is influenced by factors such as semen quality, storage conditions, and insemination timing. This study examines the effects of different semen extenders, storage durations, and insemination schedules on semen motility and pregnancy rates in Palestinian Assaf sheep. The primary aim of this study was to assess and compare the efficacy of three semen extenders – Andromed, Indonesian, and Syrian – on the quality of stored semen and its subsequent effect on pregnancy rates following AI. In addition, the study investigated the impact of semen storage duration and the timing of insemination post-equine chorionic gonadotropin (eCG) injection on reproductive outcomes.

**Materials and Methods:**

Semen was collected from eight Assaf rams, diluted with one of three extenders, and stored at 4°C for up to 72 h. Sperm motility was analyzed using a computer-assisted sperm analysis system at different time intervals. AI was performed on 180 Assaf ewes across three farms in Palestine, with insemination conducted at either 48 h, 52 h, or both 48 and 52 h post-eCG injection. Pregnancy rates were determined through ultrasound 45 days post-insemination. Statistical analysis was conducted using the Statistical Package for the Social Sciences 22 (IBM^®^ NY, USA), with p-values set at <0.05 for significance.

**Results:**

Semen motility decreased significantly over time, with total motility (TM) declining from 0.85 ± 0.01 (fresh) to 0.63 ± 0.02 after 3 days of storage. The Syrian extender exhibited superior fast motility (FM) (0.35 ± 0.01) compared to the others. Strong positive correlations were observed between TM and progressive motility (0.90, p ≤ 0.01). Pregnancy rates did not significantly differ among extenders (Andromed: 0.58 ± 0.06, Indonesian: 0.54 ± 0.07, Syrian: 0.56 ± 0.08). However, insemination performed at both 48 and 52 h post-eCG injection resulted in the highest pregnancy rate (0.62 ± 0.07), while the second ejaculation showed a tendency for improved fertility outcomes (0.61 ± 0.07).

**Conclusion:**

Prolonged semen storage negatively impacts motility, though the Syrian extender preserves FM better than the others. Pregnancy rates were not significantly influenced by the extender type but were optimized by insemination at both 48 and 52 h post-eCG injection. These findings highlight the importance of refining semen preservation techniques and timing AI procedures to enhance breeding success in Assaf sheep.

## INTRODUCTION

Livestock is a fundamental component of Palestine’s socioeconomic structure, with implications extending beyond the economy to affect ecology, environment, and cultural practices. As a vital source of income, it plays a significant role in ensuring food security for certain Bedouin and rural families [1]. Although only 13% of agricultural land is allocated for livestock purposes, this sector contributes approximately 1.7% to the national gross domestic product and accounts for 46% of agricultural income in the West Bank [2]. Given the importance of male fertility in determining the breeding potential of a flock, a comprehensive evaluation of semen parameters, sperm functionality, and routine semen evaluation has become essential [3]. Sperm motility has been reported to be positively correlated with the fertile ability of rams [4]. Recently, there has been a growing focus on the advanced evaluation of semen parameters using genomic prediction and computer-assisted sperm analysis (CASA) [5, 6]. The application of CASA allows the quantification of various sperm movement patterns, providing more detailed and accurate results than manual microscopic observation [7].

Artificial insemination (AI) is a pivotal technique for improving the genetic quality and reproductive efficiency of ewes. However, its success is influenced by various factors, including breed, age, seasonal variations, the timing of the procedure, and the use of fresh, chilled, or frozen semen, all of which impact outcomes [8, 9]. The optimal time for insemination with non-frozen semen is typically 12–18 h after the onset of estrus, and using synchronized or induced estrus can help optimize ovulation timing [10]. Different extenders, such as Tris-based solutions, are used to prepare semen for AI, and ongoing research is evaluating their effects on semen quality in various ram breeds, including kail sheep [11]. Quan *et al*. [12] have shown that Tris-based extenders effectively maintain ram sperm quality during liquid storage, although there is some decline after 24 h compared with skim milk extenders. Adding egg yolk to extenders significantly enhances sperm motility after rapid cooling and freezing [13].

In addition, previous studies by Purdy *et al*. [8] and Gaunand *et al*. [9] have emphasized the importance of timing AI for estrus detection or synchronization protocols, which significantly affect conception rates. This study examined the impact of different semen collection and insemination schedules on the quality of semen and reproductive performance of the Palestinian Assaf sheep breed using three different semen extenders. Unlike previous studies, this study integrates advanced statistical methods to identify the most relevant frequencies correlated with impedance features, providing a more comprehensive understanding. The primary aim of this study was to assess and compare the semen quality and reproductive performance of the Assaf sheep breed in Palestine using AI techniques with different timelines and semen extenders.

## MATERIALS AND METHODS

### Ethical approval and Informed consent

This study was conducted in Palestine, where there is no formal animal ethics committee currently existing. However, all experimental procedures strictly adhered to the ethical guidelines set forth by the World Organization for Animal Health, ensuring the welfare of animals used in scientific research. The research prioritized minimizing discomfort, stress, and harm to the Palestinian Assaf sheep. All procedures were supervised by experienced veterinarians and animal husbandry professionals. Farm owners provided verbal informed consent before including their animals in the study, reinforcing ethical integrity and transparency in the research.

### Study period and location

**Figure 1:**
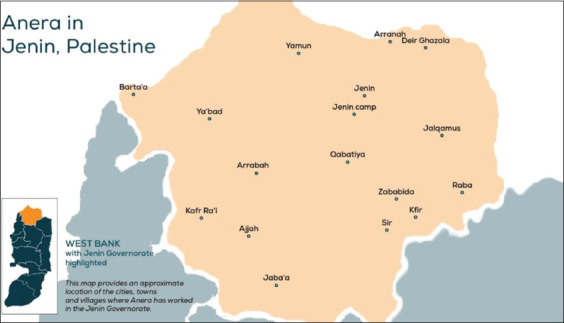
Geographic map of Jenin [Source: https://www.anera.org/stories/jenin-palestine-securing-productive-future/].

This study was conducted from March 2023 to November 2023 at four key locations in the West Bank, Palestine. The first experiment was carried out at Beit Qad Agricultural Station, a facility affiliated with the Palestinian National Agricultural Research Center in Jenin, West Bank Palestine (Coordinates: 32.4590° N, 35.3000° E) (Figure 1). The facility is a small ruminant improvement center specializing in Assaf and local Awassi sheep breeds. The second experiment was conducted on three Assaf sheep farms in Tubas Governorate, West Bank, Palestine.

### Experimental animals

Eight healthy Assaf rams aged 2–3 years were randomly selected from Beit Qad Station for the first experiment. In addition, 60 Assaf ewes were randomly selected from each farm, resulting in a total of 180 ewes for the second experiment, based on their parity records (from parity 1 to parity 4) in each farm. The dataset comprises ejaculations from 8 Assaf rams introduced to 180 Assaf ewes, detailing various aspects of AI. The variables included the type of extender used, number of ejaculations, farm, timing of insemination, ewe parity, and treatment groups. Three extenders were used: Andromed (A), 79 cases; Indonesian (I), 64 cases; and Syrian (S), 37 cases. The number of ejaculations varied, with 115, 52, and 13 ejaculations for the first, second, and third, respectively. The study involved three farms, labeled as Farm 1 (F1), Farm 2 (F2), and Farm 3 (F3), each contributing 60 ewes. Ewes were categorized into three groups based on the timing of AI relative to equine chorionic gonadotropin (eCG) injection: Group 1 (G1) for those inseminated after 48 h, Group 2 (G2) for those inseminated after 52 h, and Group 3 (G3) for those inseminated after both 48 h and 52 h, with each group consisting of 60 ewes. Ewe parity ranged from 1 to 4, with counts of 20 for parity 1, 69 for parity 2, 64 for parity 3, and 27 for parity 4. The treatments were designated as T1 through T9, representing various combinations of extender type and timing of insemination. For instance, T1 (AG1) involved ewe inseminated 48 h after eCG injection using the Andromed extender (Minitube, Germany), whereas T4 (IG1) involved ewe inseminated 48 h after injection using the Indonesian extender.

#### Experiment 1: Comparison of extenders

This experiment aimed to compare the efficiency of Andromed, Tris-egg yolk extender, and sodium citrate egg yolk in preserving sperm motility using semen from the first, second, and third ejaculates at different time intervals: 4 h post-dilution and cooling, followed by subsequent evaluations at 24, 48, and 72 h.

#### Reagents

All chemical reagents used in experiment 1 were purchased from Sigma-Aldrich, St. Louis, MO, USA, except for Andromed media from Minitube.

#### Preparation of extenders

In Palestine, the Tris-egg yolk extender is widely recognized as the “Indonesian extender,” as its formulation was initially learned from Indonesian sources. The composition adheres to the National Standardization Agency of Indonesia (SNI: 4869-1: 2017) and the Singosari AI Center protocols. Indonesian National Standard Agency [14].

To prepare the extender, 1.6% Tris(hydroxymethyl)aminomethane, 0.9% citric acid, 2.5% raffinose pentahydrate, and 1.4% lactose were dissolved in 100 mL of double-distilled water and homogenized using a magnetic stirrer for 10 min. The buffer mixture was then cooled to 37°C, after which 80 mL of the solution was combined with 20 mL of egg yolk and carefully separated from egg white using filter paper. In addition, 100,000 IU/100 mL penicillin and 0.1 g/100 mL streptomycin were incorporated. The final extender was homogenized for 30 min and refrigerated at 4°C for 24 h before use. Similarly, the sodium citrate egg yolk extender is commonly referred to in Palestine as the “Syrian extender,” as its preparation was originally adopted from Syrian sources. The formula was described by dissolving 1.94 g of glucose and 3.52 g of sodium citrate in 100 mL of double-distilled water, followed by homogenization with a magnetic stirrer for 10 min. Subsequently, 80 mL of this buffer was mixed with 20 mL of egg yolk, 0.1 g of streptomycin, and 1000 IU of penicillin G, and homogenized. The final extender was refrigerated at 4°C for 24 h before use [15, 16]. In contrast, AndroMed, a commercially available soybean lecithin-based extender produced by Minitube, was prepared according to the manufacturer’s guidelines. The preparation involved mixing one part of the AndroMed medium with four parts of double-distilled water and allowing it to stabilize for 1 h before use.

### Collection session and dilution

Three ejaculates were collected on the same day, once per week at 8:00 AM for 8 weeks, using an artificial vagina (Ref.: 11320/0000) produced by Minitube (short outer casing, small inside diameter, water temperature: 40°C–41°C). To achieve a temperature of 41°C inside the artificial vagina, water at approximately 50°C was added. However, semen collection takes time, leading to a gradual decrease in water temperature. In addition, the artificial vagina is influenced by the ambient temperature, which further contributes to the drop in temperature. The samples were immediately subjected to macroscopic and concentration evaluations. Only those meeting the standard criteria for fresh ram semen, minimum volume of 0.5 mL, wave motion score above 3 (on a 1–5 scale), minimum concentration of 2.5 × 10^9^/mL, and no abnormalities in color or odor, were used for this experiment. Each ejaculate was divided into three parts, and each part was diluted slowly using an extender at a 1:4 dilution ratio.

#### Evaluation of sperm motility parameters at different storage times

Sperm motility parameters, including total motility (TM), progressive motility (PM), circular motility (CM), fast motility (FM), local motility (LM), and Immotile (Im) were assessed with CASA system (AndroVision^®^, Minitub, Tiefenbach, Germany) to evaluate the efficiency of the different used extenders after 4 h (D0), 24 h (D1), 48 h (D2), and 72 h (D3) of dilution and cooling at 4°C in a refrigerator. Table 1 presents the distribution of semen samples according to the type of extender used, frequency of ejaculation, and duration of storage, highlighting their impact on semen preservation and quality.

**Table 1:** Distribution of semen samples by extender type, ejaculation frequency, and storage duration.

Variable	n
Extender	
AndroMed (A)	303
Indonesian (I)	149
Syrian (S)	143
Ejaculation	
First ejaculation (1)	402
Second ejaculation (2)	138
Third ejaculation (3)	55
Storage period	
Before storage (D0)	119
After 1 day of storage (D1)	216
After 2 days of storage (D2)	131
After 3 days of storage (D3)	129
Extender by storage period	
AndroMed before storage (AD0)	79
AndroMed after 1 day of storage (AD1)	106
AndroMed after 2 days of storage (AD2)	60
AndroMed after 3 days of storage (AD3)	58
Indonesian before storage (ID0)	23
Indonesian after 1-day storage (ID1)	54
Indonesian after 2 days storage (ID2)	37
Indonesian after 3 days storage (ID3)	35
Syrian before storage (SD0)	17
Syrian after 1-day storage (SD1)	56
Syrian after 2 days storage (SD2)	34
Syrian after 3 days storage (SD3)	36

#### Experiment with two different AI timelines

This experiment aimed to assess the effect of the different AI timelines on the pregnancy rate of Assaf Ewes.

#### Semen collection and dilution

Semen was collected on the same day as the insemination of eight Assaf rams. The ejaculates were evaluated by both macroscopic and microscopic methods. Only ejaculates meeting the following quality criteria were pooled and diluted with Andromed at a 1:4 ratio: a volume of at least 0.5 mL, a wave motion score above 3 (on a 1–5 scale), a concentration of 2.5 × 10^9^ sperm/mL or higher, CASA PM of 75% or greater. The diluted semen was stored at 4°C for 4 h before use.

### Estrus synchronization

Estrus was synchronized in 180 Assaf ewes selected randomly using intravaginal implant sponges (Syncro-Part^®^, CEVA, France) impregnated with 20 mg of fluorogestone acetate. The sponge remained in the vagina for 12 days, followed by an intramuscular injection of 600 IU of eCG (Folligon^®^, CEVA, France) at the time of sponge removal.

### AI

On the day of insemination using fresh semen, the ewes on each farm were randomly assigned to one of three treatment groups. Group 1 (G1) underwent a single insemination through the transcervical route 48 h after sponge removal. Group 2 (G2) underwent a single insemination through the transcervical route 52 h after sponge removal. Meanwhile, Group 3 (G3) followed a dual-insemination protocol, receiving the first insemination through the transcervical route at 48 h and the second at 52 h post-sponge removal. Each ewe was inseminated with 0.5 mL of pooled, diluted semen using an AI gun. Table 2 summarizes the AI data in Assaf ewes, comparing the effectiveness of different extenders on reproductive performance and semen quality.

**Table 2:** Data summary of artificial insemination in Assaf ewes using different extenders.

Variable	n
Extender	
AndroMed (A)	79
Indonesian (I)	64
Syrian (S)	37
Ejaculation	
First ejaculation (1)	115
Second ejaculation (2)	52
Third ejaculation (3)	13
Farm	
Farm 1(F1)	60
Farm 2 (F2)	60
Farm 3(F3)	60
Group	
Ewes artificially Inseminated after 48 h of eCG (600IU) injection (G1)	60
Ewes artificially Inseminated after 52 h of eCG (600IU) injection (G2)	60
Ewes artificially Inseminated after 48 and 52 h of eCG (600IU) injection (G3)	60
Ewe Parity	
Parity number 1 (P1)	20
Parity number 2 (P2)	69
Parity number 3 (P3)	64
Parity number 3 (P4)	27
Treatment	
Ewes artificially inseminated after 48 h of eCG injection using AndroMed extender (AG1)	20
Ewes artificially inseminated after 52 h of eCG injection using AndroMed extender (AG2)	20
Ewes artificially inseminated after 48 and 52 h of eCG injection using AndroMed extender (AG3)	20
Ewes artificially inseminated after 48 h of eCG injection using Indonesian extender (IG1)	20
Ewes artificially inseminated after 52 h of eCG injection using Indonesian extender (IG2)	20
Ewes artificially inseminated after 48 and 52 h of eCG injection using Indonesian extender (IG3)	20
Ewes artificially inseminated after 48 h of eCG injection using Syrian extender (SG1)	20
Ewes artificially inseminated after 52 h of eCG injection using Syrian extender (SG2)	20
Ewes artificially inseminated after 48 and 52 h of eCG injection using Syrian extender (SG3)	20

eCG=Equine chorionic gonadotropin

### Pregnancy status

Pregnancy status was evaluated after 45 days of insemination through a transabdominal ultrasound examination (Dawei, Wellue, China).

### Statistical analysis

All data were stored and validated using Excel sheets. Statistical analysis was performed using IBM Statistical Package for the Social Sciences 22 for Windows (IBM^®^ NY, USA), and the following models were used:

#### Model for semen evaluation

Y_ijklm_=μ + E_i_ + EJ_J_ + S_K_ + ES_l_+ e_ijkl_

Where: Y_ijkl_ denotes the observed value, while μ signifies the overall mean. The term (E_i_) represents the effect of the extender (Andromed, Indonesian, Syrian), (EJ_J_) indicates the ejaculation number (1, 2, and 3), (S_K_) reflects the effect of the storage period (D0, D1, D2, and D3), and the term ES_l_ represents the combined effect of extender type and storage period (AD1, AD2, AD3, ID1, ID2, ID3, SD1, SD2, and SD3). The term e_ijkl_ accounts for the residual error. To determine statistical significance, a threshold of (p < 0.05) was established.

#### Model of AI and pregnancy testing

Y_ijklmn_ = μ + E_i_ + EJ_J_ + F_K_ + G_L_ + P_m_ + T_n_ + e_ijklmn_

Where: Y_ijklmn_ denotes the observed value (pregnancy rate), while μ signifies the overall mean. The term (E_i_) represents the effect of the extender (Andromed, Indonesian, and Syrian); (EJ_J_) indicates the ejaculation number (1, 2, 3); (F_K_) reflects the effect of the farm (Farm 1, Farm 2, Farm 3); (G_L_) corresponds to the effect of the insemination group (Group 1, Group 2, and Group 3); (P_m_) represents the effect of ewe parity (1–4); and (T_n_) denotes the combined effect of extender type and insemination timing (T1–T9). The term (e_ijklmn_) accounts for the residual error. To determine statistical significance, a threshold of (p < 0.05) was established.

## RESULTS

### Semen evaluation

Table 3 presents p-values for fixed effect variables across the studied traits, including TM, PM, CM, FM, slow motility (SM), LM, and Im. Extender significantly affected FM and SM, with p-values of 0.02 and 0.00, respectively. Ejaculation number had a significant (p < 0.05) impact on SM. The storage period was considered a highly significant at (p < 0.001) effect across all traits. Volume significantly influenced TM, FM, and Im, with p-values of 0.02, 0.05, and 0.02, respectively. The concentration significantly affected SM with a p-value of 0.02. The interaction between the extender and storage period was highly significant (p < 0.001) across all traits, indicating a strong combined effect.

**Table 3:** Analysis of p-values for the influence of various factors on sperm motility characteristics.

Source	Trait						

	TM	PM	CM	FM	SM	LM	Im
Extender	0.80	0.70	0.47	0.02	000	0.39	0.65
Ejaculation number	0.14	0.40	0.66	0.06	0.00	0.16	0.24
Storage period	000	000	000	000	000	000	000
Volume	0.017	0.26	0.91	0.05	0.24	0.07	0.02
Concentrate	0.69	0.90	0.21	0.21	0.02	0.23	0.93
Extender by storage period	000	000	000	000	000	000	000

TM=Total motility, PM=Progressive motility, CM=Circular motility, FS=Fast motility, LM=local motility, Im=Immotile

The effects of various factors on the semen evaluation parameters are detailed in Table 4. The type of extender used, ejaculation number, and storage period significantly influenced these traits. For the extenders, no significant differences were observed in TM, PM, and CM among the Andromed (A), Indonesian (I), and Syrian (S) extenders. However, FM was significantly higher in the Syrian extender than in Andromed and Indonesian. SM was also higher in the Syrian extender group than in the other groups. Regarding the ejaculation number, no significant differences were detected in TM and PM across the three ejaculations. However, FM was significantly lower in the second and third ejaculations than in the first. The storage period had a significant impact on semen quality. TM and PM were significantly higher (p ≤ 0.05) before storage (D0) and decreased progressively over the storage period, with the lowest values observed after 3 days (D3). CM remained stable, while FM decreased significantly at D3. Im was significantly higher after 3 days of storage than in the earlier periods. The interaction between extender type and storage period revealed that TM and PM were significantly higher (p ≤ 0.05) before storage across all extenders, with notable declines after 3 days. For instance, AndroMed before storage (AD0) had TM and PM values of 0.85 ± 0.011 and 0.64 ± 0.013, respectively, which decreased after 3 days (AD3). FM and SM were significantly lower after 3 days of storage, while Im was significantly higher. These findings indicated that storage duration had a more pronounced effect on semen quality than the type of extender used or the number of ejaculation cycles.

**Table 4:** Mean ± SE of extender type, ejaculation number, and storage duration by semen evaluation parameters.

Variable	TM	PM	CM	FM	SM	LM	Im
Extender							
A	0.75^a^ ± 0.008	0.57^a^ ± 0.01	0.24^a^ ± 0.04	0.34^a^ ± 0.01	0.22^a^ ± 0.006	0.18^a^ ± 0.004	0.25^a^ ± 0.009
I	0.75^a^ ± 0.011	0.56^a^ ± 0.013	0.27^a^ ± 0.05	0.34^a^ ± 0.013	0.21^a^ ± 0.008	0.17^a^ ± 0.005	0.26^a^ ± 0.011
S	0.76^a^ ± 0.012	0.57^a^ ± 0.014	0.20^a^ ± 0.05	0.35^b^ ± 0.011	0.26^b^ ± 0.009	0.18^a^ ± 0.005	0.24^a^ ± 0.012
Ejaculation number							
1	0.76^a^ ± 0.006	0.57^a^ ± 0.007	0.27^a^ ± 0.03	0.35^a^ ± 0.008	0.22^a^ ± 0.005	0.19^a^ ± 0.003	0.24^a^ ± 0.007
2	0.74^a^ ± 0.011	0.55^a^ ± 0.012	0.24^a^ ± 0.05	0.33^b^ ± 0.019	0.21^a^ ± 0.005	0.19^ab^ ± 0.005	0.26^a^ ± 0.011
3	0.75^a^ ± 0.017	0.57^a^ ± 0.019	0.20^a^ ± 0.07	0.30^b^ ± 0.02	0.26^b^ ± 0.005	0.17^b^ ± 0.007	0.26^a^ ± 0.017
Storage period							
D0	0.85^a^ ± 0.012	0.64^a^ ± 0.011	0.42^a^ ± 0.04	0.44^a^ ± 0.012	0.21^a^ ± 0.009	0.19^a^ ± 0.005	0.16^a^ ± 0.009
D1	0.83^b^ ± 0.01	0.60^b^ ± 0.014	0.36^a^ ± 0.05	0.38^b^ ± 0.015	0.19^b^ ± 0.007	0.19^a^ ± 0.004	0.22^b^ ± 0.013
D2	0.72^c^ ± 0.012	0.55^c^ ± 0.014	0.11^a^ ± 0.05	0.28^c^ ± 0.014	0.26^c^ ± 0.009	0.17^b^ ± 0.005	0.27^c^ ± 0.012
D3	0.63^d^ ± 0.02	0.46^d^ ± 0.014	0.05^a^ ± 0.05	0.19^d^ ± 0.014	0.26^c^ ± 0.009	0.17^b^ ± 0.005	0.37^d^ ± 0.012
Extender by storage period							
AD0	0.85^ae^ ± 0.011	0.64^ad^ ± 0.013	0.43^a^ ± 0.05	0.48^a^ ± 0.014	0.20^a^ ± 0.011	0.20^a^ ± 0.006	0.15^a^ ± 0.012
AD1	0.79^ad^ ± 0.014	0.57^a^ ± 0.016	0.38^b^ ± 0.05	0.37^a^ ± 0.017	0.16^a^ ± 0.009	0.20^a^ ± 0.005	0.21^b^ ± 0.014
AD2	0.73^db^ ± 0.016	0.55^cb^ ± 0.018	0.14^b^ ± 0.06	0.29^b^ ± 0.019	0.25^b^ ± 0.012	0.17^a^ ± 0.007	0.27^bc^ ± 0.016
AD3	0.64^cb^ ± 0.016	0.46^bc^ ± 0.018	0.09^b^ ± 0.06	0.23^c^ ± 0.019	0.25^b^ ± 0.012	0.17^ab^ ± 0.007	0.35^d^ ± 0.016
ID0	0.84^e^ ± 0.026	0.66^e^ ± 0.029	0.51^c^ ± 0.10	0.47^a^ ± 0.031	0.18^a^ ± 0.019	0.16^b^ ± 0.011	0.16^a^ ± 0.026
ID1	0.83^a^ ± 0.017	0.63^a^ ± 0.019	0.49^ac^ ± 0.07	0.45^ad^ ± 0.02	0.18^a^ ± 0.013	0.20^a^ ± 0.007	0.16^ab^ ± 0.017
ID2	0.72^b^ ± 0.02	0.54^b^ ± 0.023	0.10^b^ ± 0.08	0.33^b^ ± 0.024	0.22^ab^ ± 0.015	0.17^a^ ± 0.008	0.27^cd^ ± 0.020
ID3	0.61^c^ ± 0.02	0.43^c^ ± 0.024	0.05^b^ ± 0.08	0.22^c^ ± 0.025	0.22^a^ ± 0.016	0.18^a^ ± 0.009	0.38^e^ ± 0.021
SD0	0.84^a^ ± 0.016	0.65^d^ ± 0.019	0.41^ab^ ± 0.06	0.42^a^ ± 0.02	0.24^b^ ± 0.022	0.20^a^ ± 0.012	0.16^a^ ± 0.017
SD1	0.82^a^ ± 0.03	0.61^a^ ± 0.034	0.22^b^ ± 0.12	0.37^ab^ ± 0.036	0.22^a^ ± 0.012	0.19^a^ ± 0.007	0.18^b^ ± 0.003
SD2	0.76^d^ ± 0.021	0.56^ab^ ± 0.023	0.13^b^ ± 0.08	0.29^bc^ ± 0.025	0.26^bc^ ± 0.016	0.19^a^ ± 0.009	0.24^b^ ± 0.021
SD3	0.64^c^ ± 0.02	0.45^c^ ± 0.023	0.03^b^ ± 0.08	0.18^c^ ± 0.025	0.28^c^ ± 0.015	0.18^a^ ± 0.009	0.35^de^ ± 0.021

TM: Total motility, PM: Progressive motility, CM: Circular motility, FS: Fast motility, LM: local motility, Im: Immotile. A=AndroMed, I=Indonesian, S=Syrian, D0=Before storage, D1=After 1 day of storage, D2=After 2 days storage, D3=After 3 days storage, AD0=AndroMed before storage, AD1=AndroMed after 1-day storage, AD2=AndroMed after 2 days storage, AD3=AndroMed after 3 days storage, ID0=Indonesian before storage, ID1=Indonesian after 1-day storage, ID2=Indonesian after 2 days storage, ID3=Indonesian after 3 days storage, SD0=Syrian before storage, SD1=Syrian after 1-day storage, SD2=Syrian after 2 days storage, SD3=Syrian after 3 days storage. Means in the same column with similar letters are not significantly different (p > 0.05). Covariates appearing in the model are evaluated at the following values: Volume (mL)=1.93, Conc. (Billion/mL)=3.08

### Semen parameter correlation

Table 5 presents the correlations among the various factors related to semen evaluation. The Ejaculation number showed weak correlations with other factors, such as volume and concentration, none of which were statistically significant. Volume was significantly negatively correlated (p ≤ 0.05) with total and LM and positively correlated with immotility. Concentration had a significant negative correlation (p ≤ 0.05) with SM. TM was strongly positively correlated (p ≤ 0.01) with PM and FM and moderately positively correlated with CM.

**Table 5:** Correlational analysis of semen evaluation parameters: interrelationships among ejaculation number, volume, concentration, and motility factors.

Corr.	Ejac	Vl	Conc	TM	PM	CM	FM	SM	LM	Im
Ejac.	1	−0.068	0.070	−0.022	0.0	−0.006	−0.033	0.024	−0.022	0.022
Vl		1	−0.049	−0.089*	−0.045	−0.008	−0.064	0.037	−0.082*	0.088*
Conc.			1	0.017	−0.001	0.055	0.037	−0.094*	0.054	0.004
TM				1	0.902**	0.336**	0.818**	−0.08	0.005	−0.970**
PM					1	0.318**	0.790**	0.014	−0.248**	−0.902**
CM						1	0.496**	−0.461**	−0.006	−0.318**
FM							1	−0.522**	−0.109**	−0.809**
SM								1	−0.210**	0.056
LM									1	−0.007
Im										1

Ejac=Ejaculation number, Vl=Volume, Conc=Concentration, TM=Total motility, PM=Progressive motility, CM=Circular motility, FS=Fast motility, LM=Local motility, Im: Immotile.

* Correlation is significant at the 0.05 level (2-tailed).

** Correlation is significant at the 0.01 level (2-tailed)

Furthermore, it was strongly negatively correlated (p ≤ 0.01) with immotility. PM showed strong positive correlations (p ≤ 0.01) with FM and moderate positive correlations with CM, along with a significant negative correlation with immotility (p ≤ 0.01). CM had a moderate positive correlation (p ≤ 0.01) with FM and a significant negative correlation with SM (−0.461). FM was significantly negatively correlated (p ≤ 0.01) with SM and LM and strongly negatively correlated with immotility. SM showed a significant negative correlation (p ≤ 0.01) with LM. LM had no significant correlations with other factors except for a weak negative correlation with immotility, which was not statistically significant. Immotility exhibited strong negative correlations (p ≤ 0.01) with TM, PM, and FM. Results highlighted the complex interrelationships between these factors, with several significant correlations indicating how changes in one trait may be associated with changes in others.

### AI and pregnancy rate

Table 6 presents the effects of various fixed-effect variables on pregnancy rate. Regarding the effect of each variable, no significant impact on pregnancy rate was observed for any of the examined variables.

**Table 6:** Analysis of fixed effect variables on pregnancy rate: p*-*values indicating statistical significance.

Source	p-value
Extender	0.77
Ejaculation number	0.30
Farm	0.96
Group	0.41
Parity	0.18
Treatment	0.93

The extender had a p = 0.77, indicating no significant effect. Similarly, the ejaculation number and farm displayed p = 0.30 and 0.96, respectively, both reflecting a lack of statistical significance. The group variable yielded a p = 0.41, while parity showed a p = 0.18, further confirming the absence of significant effects. Finally, the treatment variable had a p = 0.93, reinforcing the conclusion that none of the fixed-effect variables analyzed significantly influenced pregnancy rates.

Table 7 presents the mean pregnancy rates and their standard errors across different variables, including the extender type, ejaculation number, farm type, group, parity, and Treatment. The pregnancy rate was not significantly different (p > 0.05) among the extenders, with AndroMed showing a mean rate of 0.58 ± 0.06, Indonesian at 0.54 ± 0.07, and Syrian at 0.56 ± 0.08. Regarding the ejaculation number, the second ejaculation resulted in a higher pregnancy rate (not significant) of 0.61 ± 0.07 compared with the first ejaculation at 0.55 ± 0.05 and the third ejaculation at 0.36 ± 0.14. Among the farms, no significant differences were observed, with Farm 1 at 0.55 ± 0.06, Farm 2 at 0.57 ± 0.07, and Farm 3 at 0.56 ± 0.07. In terms of the group, ewes inseminated at both 48 and 52 h post-eCG injection (Group 3) had a higher (not significant) pregnancy rate of 0.62 ± 0.07 than those inseminated at either 48 h (Group 1) or 52 h (Group 2) alone, both at 0.53 ± 0.07 and 0.53 ± 0.06, respectively. Parity showed that the second and third parties had higher pregnancy (not significant) rates of 0.65 ± 0.06 compared with the first parity at 0.46 ± 0.11 and the fourth parity at 0.48 ± 0.09. For treatment, ewes inseminated using the AndroMed extender after both 48 and 52 h of eCG injection (T3) and those using the Indonesian extender under the same conditions (T6) exhibited the highest pregnancy rates of 0.70 ± 0.11. No significant differences were observed among the other treatments, with rates ranging from 0.50 ± 0.11 to 0.65 ± 0.11.

**Table 7:** Mean and pregnancy rate by extender, ejaculation number, farm, group, and treatment.

Variable	Pregnancy result		

	Mean ± SE	Lower bound	Upper bound
Extender			
A	0.58^a^ ± 0.06	0.45	0.70
I	0.54^a^ ± 0.07	0.39	0.68
S	0.56^a^ ± 0.08	0.39	0.72
Ejaculation number			
1	0.55^a^ ± 0.05	0.44	0.65
2	0.61^a^ ± 0.07	0.46	0.77
3	0.36^a^ ± 0.14	0.08	0.65
Farm			
F1	0.55^a^ ± 0.06	0.4	0.68
F2	0.57^a^ ± 0.07	0.42	0.71
F3	0.56^a^ ± 0.07	0.42	0.70
Group			
G1	0.53^a^ ± 0.07	0.38	0.67
G2	0.53^a^ ± 0.06	0.40	0.66
G3	0.62^a^ ± 0.07	0.48	0.76
Parity			
1	0.46^a^ ± 0.11	0.24	0.68
2	0.65^a^ ± 0.06	0.52	0.77
3	0.65^a^ ± 0.06	0.53	0.78
4	0.48^a^ ± 0.09	0.29	0.67
Treatment			
T1=(AG1)	0.50^a^ ± 0.11	0.28	0.719
T2=(AG2)	0.55^a^ ± 0.11	0.33	0.769
T3=(AG3)	0.70^a^ ± 0.11	0.48	0.919
T4=(IG1)	0.60^a^ ± 0.11	0.38	0.819
T5=(IG2)	0.55^a^ ± 0.11	0.33	0.769
T6=(IG3)	0.70^a^ ± 0.11	0.48	0.919
T7=(SG1)	0.65^a^ ± 0.11	0.43	0.869
T8=(SG2)	0.60^a^ ± 0.11	0.38	0.819

A=Andromed, I=Indonesian, S=Syrian, eCG=Equine chorionic gonadotropin, F1=Farm 1, F2=Farm 2, F3=Farm 3, G1=Ewes artificially Inseminated after 48 h of eCG injection, G2=Ewes artificially Inseminated after 52 h of eCG injection, G3=Ewes artificially Inseminated after 48 and 52 h of eCG injection. T1 (AG1)= Ewes artificially inseminated after 48 h of eCG injection using AndroMed extender; T2(AG2)=Ewes artificially inseminated after 52 h of eCG injection using AndroMed extender, T3(AG3)=Ewes artificially inseminated after 48 and 52 h of eCG injection using AndroMed extender, T4(IG1)=Ewes artificially inseminated after 48 h of eCG injection using Indonesian extender, T5(IG2)=Ewes artificially inseminated after 52 h of eCG injection using Indonesian extender; T6(IG3)=Ewes artificially inseminated after 48 and 52 h of eCG injection using Indonesian extender, T7(SG1)=Ewes artificially inseminated after 48 h of eCG injection using Syrian extender, T8(SG2)=Ewes artificially inseminated after 52 h of eCG injection using Syrian extender, T9(SG3)=Ewes artificially inseminated after 48 and 52 h of eCG injection using Syrian extender. Means in the same column with similar letters are not significantly different (p>0.05)

## Discussion

### Semen evaluation

This study observed variations in semen evaluation parameters influenced by different factors. The type of extender, ejaculation number, and storage period significantly affected these traits. Semen motility parameters such as TM, PM, and CM did not show significant differences across the Andromed, Indonesian, and Syrian extenders, which is consistent with a previous study by Rajashri *et al*. [17]. However, the Syrian extender exhibited higher fast and SM, suggesting that although basic motility parameters may remain unchanged, certain extenders can enhance specific motility traits due to their unique composition, as discussed by Arando *et al*. [18].

Regarding ejaculation number, no significant differences were detected in total and PM across the three ejaculations, consistent with David *et al*. [19], who reported consistent motility levels in the initial ejaculations. However, a decrease in FM in the second and third ejaculations compared with the first suggests a potential decline in semen quality with successive ejaculations, possibly due to reduced sperm concentration or energy reserves, as noted by Salamon [20].

The storage period had a pronounced impact on semen quality, with total and PM significantly higher before storage and decreasing over time. This is consistent with the findings of Joshi *et al*. [21], who highlighted the detrimental effects of prolonged storage on sperm motility and viability. The stability of CM and the significant decrease in FM during storage emphasize the importance of minimizing storage time to preserve semen quality. The increase in immotility after 3 days of storage corroborates with Fernandes *et al*. [22], who observed similar trends in stored semen samples.

The interaction between extender type and storage period revealed that total and PM were significantly higher before storage across all extenders, with notable declines after 3 days. This suggests that although extenders may initially preserve motility, their protective effects diminish over time, as also reported by Paulenz *et al*. [23]. The significant decreases in fast and SM after 3 days of storage, along with the increase in immotility, underscore the critical role of storage duration in semen preservation [24].

Optimizing storage conditions and minimizing storage duration is crucial for maintaining semen viability, a conclusion supported by multiple studies in the field [2, 22]. These insights will guide future research into semen preservation and AI programs.

### Semen parameter correlation

The correlation analysis conducted in this study revealed a strong positive correlation between TM and PM (p ≤ 0.01), consistent with the findings of Sinapov and Yotov [25] regarding the relationship between various kinematic parameters of ram semen and the reproductive performance of dairy sheep following assisted reproductive technologies. Specifically, TM exhibited a significant positive correlation with PM. Conversely, while Sinapov and Yotov [25] did not report significant differences in ejaculate volume concerning reproductive performance, the present study identified a significant negative correlation between Volume and TM (−0.089, p ≤ 0.05). This suggests that although ejaculate volume may not directly influence reproductive outcomes, it may still affect motility parameters under varying conditions. Furthermore, a negative correlation was observed between immotility and the other motility parameters, which is consistent with a previous study by Sinapov and Yotov [25] that established a strong negative relationship between TM and Immotility. This finding reinforces the notion that higher motility is associated with lower immotility, which is a critical factor in the evaluation of semen quality.

Our results underscore the essential role of kinematic parameters in assessing semen quality and reproductive performance. The strong correlations identified between TM, PM, and immotility highlight their significance in successful insemination outcomes. However, the differences in the significance of volume and its relationship with motility parameters indicate that further investigation is warranted to fully understand the complexities involved in semen evaluation.

### AI and pregnancy rate

The analysis of pregnancy rates across different variables (Table 7) provides several insights into the factors influencing reproductive success in sheep breeding. The comparison of pregnancy rates among different extenders, such as AndroMed, Indonesian, and Syrian, revealed no significant differences. This observation is consistent with the studies by Fiser and Fairfull [26] and Quan *et al*. [27], which emphasize the role of extender composition in maintaining sperm motility and viability. Although the differences were not statistically significant, the slight variations suggest that the formulation of extenders can influence reproductive outcomes.

Regarding the ejaculation number, the second ejaculation tended to result in a higher pregnancy rate than the first and third ejaculations, although these differences were not statistically significant. This trend is consistent with the findings of Gibbons *et al*. [28] and Gunter *et al*. [29], who highlighted the impact of physiological maturity and experience on reproductive success. The higher rate of the second ejaculation may reflect the optimal quality and quantity of sperm.

No significant differences in pregnancy rates were observed among the farms in terms of farm-specific factors. This suggests that management practices and environmental conditions are consistent across farms, as noted by Wrench *et al*. [30] and Houghton *et al*. [31]. The uniformity of rates indicates that farm-specific factors are well-managed, leading to similar reproductive outcomes.

The timing of insemination also plays a crucial role, with ewes inseminated at both 48 and 52 h post-eCG injection showing a higher pregnancy rate compared to those inseminated at either 48 or 52 h alone. This finding supports the findings of Kumar *et al*. [32] and Menchaca *et al*. [33], which emphasize the importance of precise timing in AI success. The combined timing may optimize the physiological readiness of the ewes for conception.

Parity was another factor influencing pregnancy rates, with the second and third parity showing higher rates than the first and fourth parity. This observation is consistent with the studies by Gibbons *et al*. [28] and Gunter *et al*. [29], who suggested that ewes with more maturity and experience have better reproductive outcomes.

Finally, treatment involving the use of specific extenders, such as AndroMed and Indonesian, after both 48 and 52 h of eCG injection resulted in the highest pregnancy rates. This supports the findings of Hackett *et al*. [34], who noted the positive impact of eCG on pregnancy rates. The combination of specific extenders and timing enhances reproductive success.

## CONCLUSION

This study assessed the impact of different semen extenders, storage durations, and insemination schedules on semen quality and reproductive performance in Palestinian Assaf sheep. The findings underscore the significance of storage duration on sperm viability, with TM declining significantly from 0.85 ± 0.01 (fresh) to 0.63 ± 0.02 after 3 days of storage. Among the tested extenders, the Syrian extender demonstrated superior FM (0.35 ± 0.01), yet pregnancy rates remained statistically comparable across extenders (Andromed: 0.58 ± 0.06, Indonesian: 0.54 ± 0.07, Syrian: 0.56 ± 0.08). Notably, insemination at both 48 and 52 h post-eCG injection yielded the highest pregnancy rate (0.62 ± 0.07), suggesting a potential optimization strategy for AI programs.

One of the key strengths of this study is its comprehensive evaluation of semen extenders under real farm conditions, providing insights into optimal semen preservation and insemination timing strategies. The use of CASA enhanced the precision of semen quality assessments, reducing human variability in motility analysis. In addition, the study’s design, encompassing multiple farms and controlled insemination schedules, increases the generalizability of the results within the Palestinian Assaf sheep breed.

Despite these strengths, certain limitations must be acknowledged. First, while semen motility is a crucial indicator of fertility, other parameters such as sperm morphology and membrane integrity were not assessed, which could provide a more holistic understanding of sperm viability. Second, the study was confined to a specific breed and regional climatic conditions, which may limit extrapolation to other sheep breeds or geographic regions with different environmental conditions. Furthermore, although pregnancy rates were measured, lambing rates and offspring viability were not evaluated, which could have provided further insights into the long-term reproductive efficiency of AI practices.

Future research should explore the incorporation of advanced sperm preservation techniques, such as antioxidants or cryoprotectants, to mitigate the detrimental effects of prolonged storage. In addition, studies evaluating AI success in different breeds under diverse environmental conditions would enhance the applicability of findings. Investigating genetic and physiological factors influencing fertility outcomes, alongside AI efficiency in field conditions, could further improve reproductive strategies for Assaf sheep and other livestock breeds.

This study highlights the importance of optimizing semen storage and AI timing to maximize fertility rates in Assaf sheep. While semen extenders influence motility, insemination strategy remains a key determinant of reproductive success. Continued advancements in AI protocols will be crucial in enhancing breeding efficiency and genetic improvement in sheep farming systems.

## AUTHORS’ CONTRIBUTIONS

WH, IBS, and NM: Planned and coordinated the study and prepared the final version of the manuscript. WH, SK, and BB: Conducted the analyses, performed the statistical analysis, and participated in preparing the final version of the manuscript. HC and MS: Participated in the analyses and wrote the first draft of the manuscript. WH, IBS, and BB: Participated in the analyses and data archiving. All authors have read and approved the final manuscript.
